# Physiological Antagonism in the Treatment of Fever

**Published:** 1876-12

**Authors:** E. T. Easley

**Affiliations:** Little Rock, Ark.


### PHYSIOLOGICAL ANTAGONISM IN THE TREATMENT OF FEVER.

BY E. T. EASLEY, A. M., M. D., LITTLE ROCK, ARK.

The old pathologists insisted upon regarding fever as an entity. They busied themselves to find the organs or parts chiefly affected, and to such a nidus, most supposititious, they made haste to trace all its phenomena. The therapeutics based upon this idea were very far from representing a rational medication. As a necessary consequence, great epidemics, in which the inflammations were conspicuous factors, were largely fatal, and it could hardly be otherwise, for a treatment founded upon false premises as to the morbid condition, was worse in its relation to the individual than the stupidity of empiricism. It is to be observed, however, that there is this difference between a strictly empirical mode of procedure and one resulting from erroneous conclusions. The former is purely guess work, whereas the latter betrays an effort and anxiety to attain the truth. Although we may be groping in the dark, if we work with singleness of purpose and desire earnestly to see the light, we will undoubtedly make progress. Such workers, indeed, continually declare:

> "'Tis sometime yet

> To the gray dawning, but we move betimes,

> And our impatience ushers in the day."

Advances in this direction were exceedingly slow, step by step was made the heavy march through the mire, until the granite was achieved. Microscopic workers build up vast islands in the bosom of the mighty deep; drop by drop, even slower, the stalagmites and stalactites of the pillared caves grow big and strong and beautiful. In this way, on the natural principle of the survival of the fittest, have great systems been built up from the shining gold of many an imperishable thought, sifted from the *debris* of dogmas overthrown.

The difficulty of our predecessors has been stated in the beginning, and if we have emerged from it, it is because we begin where they left off, and refuse to retrace the labyrinths in which they wandered. But let no one be deceived, or suppose that all the problems of the phlegmasiæ are explained. Many of them remain unsolved, albeit we have brought to bear upon them dissection, the microscope, clinical observation and the modern inductive reasoning. We avoid the grosser mistakes, but as our investigations widen we are constantly reminded of how much there is past our finding out. We have learned notably a great deal about epidemic constitution, and the natural history of disease. No medical man would now be so ignorant as to propose to cure in a few hours or days a malady with a determinate course, as for example small pox or typhoid fever. It is only the very silly or dishonest who make such vaunts.

As late as the introduction of the cinchona barks, when it was demanded of a celebrated quack, "*Quid est febris?*" he replied with unusal honesty, (for these people know everything and perform prodigies) "I do not know," and until this hour we have failed in a definition precise enough and sufficiently comprehensive. But we understand the signs of fever, at any rate recognize them, and are in the habit of making an interpretation of its phenomena. We give to a set of symptoms—acceleration of pulse, increased heat of surface, pain and suspension or perversion of glandular action, individually absent or more or less prominent, the name *fever*. Notwithstanding we have yet much to learn, we have acquired an earlier recognition of symptoms, and a wiser appreciation of their meaning. Probably the most useful conception we have reached, clinically considered, is that embraced in the subject of this paper—the principle of physiological antagonism in the treatment of fever. To this proposition may be added, as pertinent to our own country since the introduction of quinine, the vast importance attached to the prophylaxis.

It has been demonstrated that an antagonism of action exists between remedies and diseased states, similar to that which is known to obtain between drugs themselves. Dr. Roberts Bartholow has in particular, (vol. 2, No. 1, *American Clinical Lectures*,) directed attention to the subject, and his reasoning is so just and accurate that I make no apology for the large use now made of his conclusions.

In a practical enquiry of this sort we are to consider what means are at our command to antagonize the morbid processes, and are to have something like a definite conception of what is comprehended in the complexus of diseased action, which we designate fever. For the purposes of this paper, fever signifies an increment of the body-heat—an exaltation of temperature—an exaggeration of the combustion process. The tendency of a high degree of pyrexia can only be toward death. In this condition of preternatural body-heat there occurs not only a functional weakening, (parenchymatous degeneration of the Germans) but a positive loss of substance is sustained. It is also not to be forgotten that with rapid waste of nitrogenous tissue, there is combined suspension of glandular action and retention of excrementitious matters. A temperature abnormally elevated cannot exist without functional derangement of important organs. The necessity for reducing temperature is, therefore, one which demands our earliest and most persistent efforts, an indication which we cannot fail to see, and which constantly intrudes itself upon us. To enable the body with the least expenditure of force to tide over its period of extra work, to modify and restrain the general process of combustion which is taking place in excess, must be our object. Now, as these changes occur in proportion to the degree of the fever heat, it is a just deduction that temperature is the chief factor in their production. Our countryman, Dr. H. C. Wood, (*a Study of Fever—Toner Lecture*) concludes that external heat applied to the body, so as to elevate the temperature, produces derangement of function exactly similar to that which is observed in natural fever, "the intensity of the disturbance being directly proportionate to the rise in temperature." It is an old observation in vivisection, lately repeated by Brunton, Bartholow and myself, that heat applied to the organ causes an increase in the number of heart-beats, and this augmentation of the contractions bears a progressive relation to the amount of heat applied, until the viscus has reached its highest rate of speed. Conversely, it has been very easy to show in these experiments that cold has precisely an opposite effect. The destructive results of continued high temperature can hardly be overestimated, and from this follows plainly enough the proposition that the most urgent indication of treatment is to *diminish the unnatural heat.* The problem, as stated, is to abate heat, and no measure can be of more obvious utility than the use of cold. It is wonderful that men's minds were so enthralled by dogmas that the simple and natural employment of this agent was neglected. Since, however, we have learned so much from the the thermometric study of temperature, a scientific application of the method may be made, and having passed from the hands of empirics, it can no longer be ignored. We mean that the thermometer and sphygmograph, and the elaborate investigations of the subject, have placed the use of cold water as a remedy in pyrexia upon an ascertained foundation, a scientific basis.

As early as the close of the last century, Nathan Smith, of this country, and Currie of Liverpool, began to insist upon a rational use of cold water, but it was reserved for our friends across the water, and that quite recently, to systematize the practice. The effect of an external lowering of temperature is to lessen the frequency of the cardiac impulses, and to limit those damaging tissue changes produced by preternatural heat. "It is simply," says Roberts Bartholow, "a question *cæteris paribus* of the degree of heat and the amount of cooling necessary to abate it." Now, as to the value of this therapeusis, we know that when a fever patient, with a temperature of 100° to 105° is placed in a bath, and the thermometer falls to 98° or lower, that the unnatural surface heat is abstracted, and that the blood is cooled in the capillary vessels. We comprehend that good has been done, that the morbid processes have at least been interrupted, and that comfort is secured to the patient, suffering from the most exhausting and devitalizing of all diseased actions. This is the practical, common sense view of the matter, and receives a beautiful illustration in one of Dr. Woods' recent experiments, for this distinguished observer discovered that an animal heated in a hot-air chamber until the febrile state was induced, became restored to the normal condition by being plunged into a cold bath. The need of an early reduction of exalted temperature will be apparent when we consider its disastrous effects upon the heart itself. The classical alterations gathered from many autopsies are that the organ is much diminished in consistence, its softened tissue is readily torn and broken down, its color pale, and the ventricular walls in most cases lessened in thickness. That these deadly changes are the result of persistent high temperature, over-work and impaired nutrition, is too manifest to require illustration. I have elsewhere undertaken to show the eminent danger of heart failure in long continued pyrexia. I only add now on that subject, and as bearing directly upon our present enquiry, the voluminous language of Juergensen: "It must never be forgotten that the most dangerous enemy to the heart is the high temperature, and that this may be safely and quickly lowered by bathing."

In regard to the manner of using cold, and the special indications for its employment, only a few general statements can be required, as every one is supposed to be familiar with the few contra-indications. Practically, unless the collapse be extreme, the direct abstraction of heat will in most cases be very satisfactory, if care be used in the administration of stimulants, and in regulating, by the thermometer range of the patient, the temperature of the baths.

When we speak of a cold bath, it is to be remembered that a degree of cold relative to the temperature of the patient is meant, and this is an important point. For instance, in an increment of body heat of 4°, with a thermometer registry of 102°, great comfort and relief will be experienced by the use of water at 98°, or the normal body heat. In this way the exalted temperature of fever may be *gradually* reduced by repeated bathings to a desirable degree.

Enough has, perhaps, been said to show that we have no means so direct, immediate and attainable to reduce abnormal heat as cold—in a word, that it is the natural and simplest antagonist of the febrile state. It is an agent always available, and at the command of the provincial practitioner, and its good results are immediately apparent.

We are, however, in many inflammatory affections, restricted to the use of anti-pyretic medicines, such agents as quinine, digitalis, aconite and veratrum veride. These depress temperature, also; there are special indications for the use of each of them, and their value as physiological opponents of the febrile state will constitute the subject of another paper.

